# Nanocrystals of Mangiferin Using Design Expert: Preparation, Characterization, and Pharmacokinetic Evaluation

**DOI:** 10.3390/molecules28155918

**Published:** 2023-08-07

**Authors:** Abdur Rehman Sarwar, Furqan Muhammad Iqbal, Muhammad Anjum Jamil, Khizar Abbas

**Affiliations:** 1 Department of Pharmaceutics, Faculty of Pharmacy, Bahauddin Zakariya University, Multan 60800, Pakistan; arehmanchandio@yahoo.com (A.R.S.); raoanjumjamil@gmail.com (M.A.J.); 2 Department of Pharmacognosy, Faculty of Pharmacy, Bahauddin Zakariya University, Multan 60800, Pakistan

**Keywords:** mangiferin, nanocrystals, in vitro release, pharmacokinetics

## Abstract

Making nanoscale drug carriers could boost the bioavailability of medications that are slightly water soluble. One of the most promising approaches for enhancing the chemical stability and bioavailability of a variety of therapeutic medicines is liquid nanocrystal technology. This study aimed to prepare nanocrystals of mangiferin for sustained drug delivery and enhance the pharmacokinetic profile of the drug. The fractional factorial design (FFD) was used via a selection of independent and dependent variables. The selected factors were the concentration of mangiferin (A), hydroxypropyl methyl cellulose (HPMC) (B), pluronic acid (C), tween 80 (D), and the ratio of antisolvent to solvent (E). The selected responses were the particle size, polydispersity index (PDI), zeta potential, and entrapment efficiency. The nanocrystals were further evaluated for mangiferin release, release kinetics, Fourier transforms infrared spectroscopy (FTIR), differential scanning calorimetry (DSC), X-ray diffraction (XRD), particle size, zeta potential, and scanning electron microscopy (SEM). The stability studies of developed nanocrystals were performed for 6 months and pharmacokinetics on albino rabbits. The value of entrapment efficiencies ranged from 23.98% to 86.23%. The percentage release of mangiferin varied from 62.45 to 99.02%. FTIR and DSC studies showed the stability of mangiferin in the nanocrystals. The particle size of the optimized formulation was almost 100 nm and −12 mV the value of the zeta potential. The results of stability studies showed that the nanocrystals of mangiferin were stable for a period of six months. The peak plasma concentration of mangiferin from nanocrystals and suspension of mangiferin were 412 and 367 ng/mL, respectively. The value of AUC_0−t_ of nanocrystals and suspension of mangiferin was 23,567.45 ± 10.876 and 18,976.12 ± 9.765 µg×h/mL, respectively, indicating that the nanocrystals of mangiferin showed greater availability of mangiferin compared to the suspension of the formulation. The developed nanocrystals showed a good release pattern of mangiferin, better stability studies, and enhanced the pharmacokinetics of the drug.

## 1. Introduction

Currently, over 40% of medicines in expansion lines are poorly water soluble, and up to 60% of molecules immediately from synthesis are also poorly soluble. In most circumstances, low solubility is related to poor bioavailability [1]. Improvement in solubility and increasing the rate of dissolution are fundamental techniques concerned with the medication bioavailability issues of drugs [2]. Micronization, which results in mean particle sizes of roughly 3–5 μm, is a simple approach for improving the dissolving velocity by increasing the surface area of the powdered drug. However, because many of the novel chemicals have limited solubility, micronization does not result in a significant improvement in bioavailability following oral administration [3]. The greatest difficulty for pharmaceutical development is to develop innovative formulation processes and drug delivery systems to solve the drug molecules’ solubility issues, which are frequently linked to low oral bioavailability [4]. Nanotechnology is already attracting the attention of medicinal experts [5]. The bioavailability of weakly water-soluble pharmaceuticals might be increased by adopting the approach of making nanoscale drug carriers. Liquid nanocrystal technology is one of the most promising methods for improving the bioavailability and chemical stability of a wide range of medicinal agents. Any nanomaterial with a particle size of 100 nm that is single crystalline is referred to as a nanocrystal. The phrase “drug nanocrystals” suggests that the isolated particles are crystalline; however, they can alternatively be partly or fully amorphous, depending on the manufacturing procedure [6]. Drug nanocrystals should be separated from polymeric nanoparticles, which are composed of a polymeric matrix and a drug that has been integrated. Drug nanocrystals are free of any matrix substance.

Bioactive chemicals are plentiful in fruits, vegetables, grains, pulses, roots, and other plant sources. Natural bioactive chemicals, on the other hand, are unstable in nature chemically and prone to destruction through oxidative processes when exposed to light, oxygen, heat, and moisture. The destruction of compounds that are phenolic in nature through the oxidative process may lead to the configuration of free radicals and the growth of a distasteful taste and unpleasant smell in the equipped product, which may have a detrimental impact on shelf life, sensory qualities, and customer acceptance [7]. Furthermore, the use of pure bioactive substances in biological formulations is relatively limited due to several characteristics, such as release in a fast manner, very low solubility, less bioavailability, and the potential to disintegrate in the presence of environmental conditions. Encapsulation is thus seen as a viable option for preserving the bioactive quality ingredients or increasing their application in food, nutraceuticals, or compositions in a biological manner. Microencapsulation and nano-encapsulation are the two foremost techniques in encapsulation technology, with both specializing in improving product functionality [8]. The use of nano-encapsulation techniques for the delivery of natural compounds is increasing day by day.

A naturally occurring bioactive and poly-phenol substance can be extracted from the bark of the stem and leaf of the mango tree (*Mangifera indica*). In Pakistan, mango trees are numerous [9]. Mangiferin is stable under heat and likely pharmacologically dynamically bioactive with anti-inflammation, anti-infectious, immunomodulatory, anti-tumor, cardiovascular effects, anti-atherosclerotic, and antioxidant properties. Mangiferin has been proposed as a possible neuropharmacotherapeutic [10]. Mangiferin reduces the mediators of inflammation in human beings and other animals, suggesting its anti-inflammatory properties. These properties were confirmed by various tests. Mangiferin clearly has pharmacological significance. Thoughtful the fundamental phenomenon of Mangiferin activity allows designed for the logical creation of novel preventative and therapeutic strategies [11]. Many researchers have reported microspheres [12], nanoparticles [13], and many other dosage forms for the delivery of mangiferin. These systems deliver mangiferin for the management of infections, tumors, hypertension, and atherosclerosis. These researchers showed that the pharmacokinetics of mangiferin is very low from already developed systems. The nanocrystals are developed to improve the pharmacokinetics of mangiferin. The nanocrystals are easily developed within a short time and are stable, and that is why nanocrystals are prepared in this study.

HPMC K15M is easily soluble in water and possesses better film-forming properties because of its hydrocolloid-forming behavior [11]. Substituting the degree pattern, presence of the functional group, and chain length of polymeric materials have a significant impact on the permeability, solubility, and mechanical property. HPMC is the most common hydrophilic vehicle employed in the development of orally controlled drug delivery systems. Many complicated and significant mechanisms are involved in the release of the active moiety from developed micro- and nano-carriers developed by using HPMC K15M utilizing waster as solvent. When HPMC comes into contact with gastrointestinal fluid, it swells significantly and eventually dissolves [14]. Different parameters with the intention of a system that can impact the release of properties of an active moiety from the matrix have previously been explored and reported [15].

Pluronic F127 is made up of poly-oxyethylene units (70%) and poly-oxypropylene blocks (30%). When Pluronic F127 is present in an aqueous solution at a concentration of 15% or more, it changes starting from a barely viscous to semi-solid gel with heating from 4 °C to 23 °C, and this thermo-gelation is reversed upon cooling. A sol–gel transition temperature characterizes the thermogelling phenomena. That is, below this temperature, the sample is liquefied, allowing for a pleasant and accurate administration; beyond this temperature, the solution gels as the local temperature rises. The interaction of the various Pluronic molecules causes thermogelification. The increase in temperature alters the hydration spheres roughly the hydrophobic units, which results in stronger communications between these hydrophobic units [16]. Pluronic F127 is employed in various drug delivery carriers and extensively used for the formation of micelles. These micelles forming properties of pluronic F127 have the ability to solubilize the drugs that are poorly water-soluble. The drugs that are compatible with the core of the micelles are suitable for delivery to the target site using Pluronic F127 as a polymer. The compatibility of the active moiety with the central part of the micelle is an important aspect of drug solubilization. Pluronics’ solubilization capabilities have led to a wide range of uses, particularly in pharmaceutics and medicine. Pluronics’ capacity to micellar solubilize has stimulated great interest in the delivery of drugs. Pluronics are widely deemed secure and non-hazardous for administration orally and parenterally, and the FDA has authorized their pharmaceutical and medical uses [17]. Tween 80 has high membrane fluidity associated with a low phase-transition temperature. It is used as an emulsifier, solubilizer, stabilizer, softener, anti-static agent, etc. Tween 80 has been used in various studies to assess its effect on different drug-carrying vesicles [18]. The tween 80 effectively inhibits the p glycoproteins that are involved in the efflux of many drugs, which causes the ineffectiveness of the majority of drugs.

The present study aimed to develop nanocrystals of mangiferin using factorial design. The nanocrystals were characterized using FTIR, DSC, and XRD, particle size, zeta potential, and SEM. An HPLC method was developed for the estimation of mangiferin, and stability studies were also performed on the optimized formulation. The pharmacokinetics of mangiferin were studied using albino rabbits.

## 2. Results and Discussion

### 2.1. Preformulation Study

Mangiferin melts at 278–280 °C, which was previously described in the literature [19]. The purity of a drug is determined by its melting point. If the melting temperature of the drug shows variation, then impurities may be present. The given sample of mangiferin melts at the mentioned temperature, which confirms that the provided sample is mangiferin. Khurana et al., in 2017, studied the physical properties and similar melting points of mangiferin [20]. The melting point and solubility studies were successfully performed on mangiferin. Mangiferin melted between 278 and 280 °C, which confirmed its purity. This study also reports similar findings on the melting point of mangiferin. In all solvents studied, solubility increases with the increase in temperature. The highest values of solubility of mangiferin were obtained with ethanol and methanol, and the smallest values were obtained using n-hexane. An ANOVA test indicated that there was a significant difference between ethanol and methanol with water, ether, acetone, and n-hexane at the temperature ranges of 5 °C and 150 °C. In the case of 30 °C and 450 °C, hexane, acetone, and ether were significantly different from water, methanol, and ethanol, and for the temperature of 600 °C, all the solvents studied were significantly different [21,22]. The solubility of mangiferin increased with the increase in temperature. The maximum solubility of mangiferin was observed with ethanol and methanol, and the smallest values were obtained using n-hexane. Wu et al. in 2021 also studied similar findings of the solubility of mangiferin [23].

### 2.2. Preparation of Nanocrystals and Statistical Analysis of Experimental Data Using Design-Expert

Nanocrystals were prepared successfully and evaluated for different parameters via using the experimental design. Some intricate experimental designs, like the factorial design that use a response surface methodology, can be optimized. These studies try to relate variables and experimental results as effectively as possible. Utilizing a factorial approach, the composition of mangiferin was optimized to identify the best formulation variables that influence product quality. The four responses were observed during the use of experimental design [24], as mentioned in Table 1. The particle size of the developed formulations ranged from 418 nm to 620 nm, and the PDI ranged from 0.428 to 0.787. The PDI values showed a variety of particle sizes in the developed formulation. The F6 formulation showed the lowest particle size, while F12 showed the maximum particle size. The value of zeta potential lies in the negative range. The F10 formulation showed −13 mV zeta potential values, which was the maximum value as compared to the other formulations. The negative values of zeta potential showed more stability of the formulation as compared to the positive values. The value of entrapment efficiencies ranged from 23.98% to 86.23%. The F2 formulation showed the minimum entrapment efficiency of mangiferin, whiles the F3 formulation showed maximum values.

#### 2.2.1. Analysis of Particle Size

**Figure 1:**
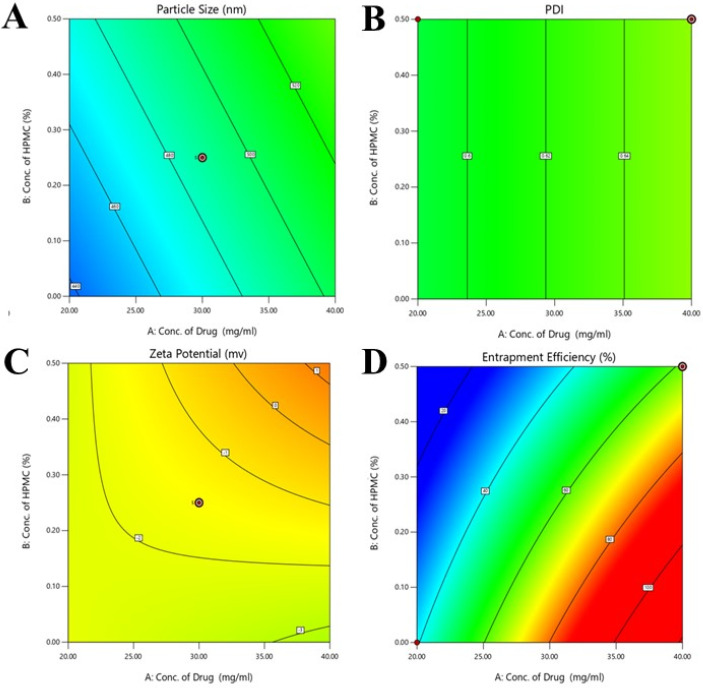
Contour graphs (**A**–**D**) showing the effects of concentration of polymers and drugs on the particle size, PDI, zeta potential, and entrapment efficiency of the developed formulations. In graphs the different color appears due to the use of design expert software. In (**A**–**D**) the concentration of HPMC and drug are 0.00 to 0.50 % and 20.00 to 40.00 % respectively. In (**A**) particle size value ranged from 46 to 52 nm and PDI value ranged from 0.60 to 0.64 in (**B**). In (**C**) value of zeta potential ranged from −2 to 1 and entrapment efficiencies ranged from 20 to 100 % in (**D**).

**Figure 2:**
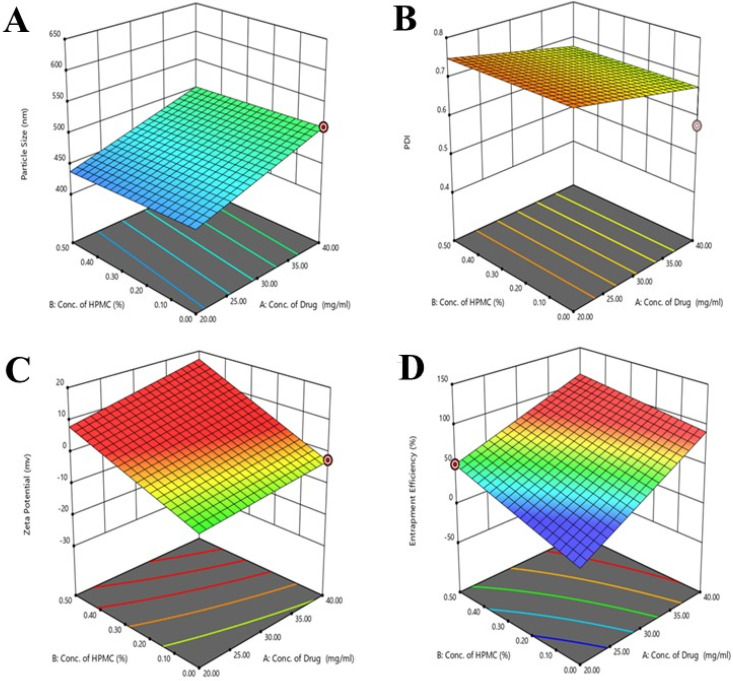
The 3D graphs (**A**–**D**) showing the effects of concentration of polymers and drugs on the particle size PDI, zeta potential, and entrapment efficiency of the developed formulations. In graphs the different color appears due to the use of design expert software.

The particle size of the developed formulations was evaluated by the experimental design [25]. The contour and 3D graphs are shown in Figure 1 and Figure 2, respectively. The ANOVA results showed that the value of *p* was 0.0310, indicating the significance of the model because the *p* value was less than 0.05. In the lack of fit value, the *p* value was greater than 0.05, indicating the non-significance of results. The value of *f* was 3.84 in the case of the applied model. The concentration of the drug and HPMC showed different effects on the particle size of nanocrystals, as mentioned in contour and 3D Figure 1A. When the concentration of the drug decreased in the formulation of nanocrystals, the particle size decreased, and the particle size increased when the concentration of HPMC decreased, as mentioned in 3D graphs. When the concentration of pluronic acid increased, the particle size of the nanocrystals increased, and an increased concentration of tween 80 produced small size particles, which can effectively deliver the drug in a controlled manner.

#### 2.2.2. Analysis of PDI

The ANOVA results showed that the value of *p* was 0.0526, indicating the significance of the model because the *p* value was less than 0.05. In lack of fit value, the *p* value was less than 0.05, indicating the significance of the results. The *p* value for the concentration of the drug, Pluronic acid, volume ratio of solvent, and anti-solvent was also less than 0.05, indicating the significance of the results. The value of *f* was 3.20 in the case of the applied model. The concentration of the drug and HPMC showed different effects on the PDI of developed nanocrystals, as mentioned in interaction, predicted versus actual, contour, and 3D graphs. When the concentration of the drug decreased in the formulation of nanocrystals, the PDI decreased, and the PDI increased when the concentration of HPMC decreased, as mentioned in 3D graphs. The increased concentration of pluronic acid and tween 80 in nanocrystals formulation showed a decreased value of PDI. Systems with droplets that have a uniform distribution have lower PDI values. Pluronic acid and tween 80 were discovered to be significantly effective in the model when the PDI responses for nanocrystal formulation were investigated. Also, due to the linearity of the model, the program did not produce results for binary interactions. The independent factors’ surface and contour graphs show a statistically significant impact on PDI responses.

#### 2.2.3. Analysis of Zeta Potential

The ANOVA results showed that the value of *p* was 0.0021, indicating the significance of the model because the *p* value was less than 0.05. In the lack of fit value, the *p* value was greater than 0.05, indicating the non-significance of results. The *p* value for the concentration of tween 80, Pluronic acid, and volume ratio of the solvent and anti-solvent was also less than 0.05, indicating the significance of the results. The value of *f* was 19.66 in the case of the applied model. The concentration of the drug and HPMC showed different effects on the zeta potential of developed nanocrystals, as mentioned in interaction, predicted versus actual, contour, and 3D graphs. When the concentration of the drug decreased in the formulation of nanocrystals, the value of zeta potential increased towards a negative value, and the zeta potential value decreased when the concentration of HPMC decreased, as mentioned in 3D graphs.

#### 2.2.4. Analysis of Entrapment Efficiency

The ANOVA results showed that the value of *p* was less than 0.0001, indicating the significance of the model because the *p* value was less than 0.05. In the lack of fit value, the *p* value was less than 0.05, indicating the significance of the results. The *p* value for the concentration of tween 80, Pluronic acid, and volume ratio of solvent and anti-solvent was also less than 0.05, indicating the significance of the results. The value of *f* was 231.90 in the case of the applied model. The concentration of the drug and HPMC showed different effects on the entrapment efficiencies of developed nanocrystals, as mentioned in interaction, predicted versus actual, contour, and 3D graphs. The value of entrapment efficiencies decreased when the concentration of mangiferin in the nanocrystals decreased. When the concentration of pluronic acid increased, the entrapment efficiency of mangiferin increased.

### 2.3. In Vitro Mangiferin Release

**Figure 3:**
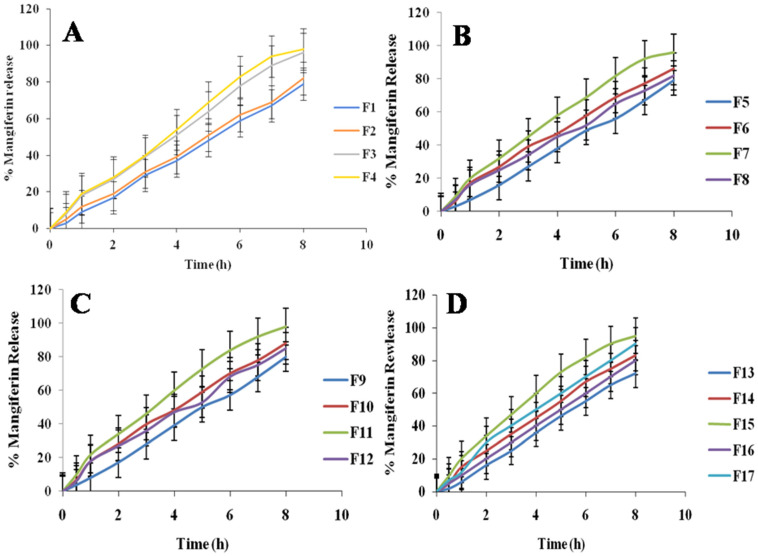
Mangiferin release from prepared nanocrystals of formulations F1 to F4 (**A**), F5 to F8 (**B**), F9 to F12 (**C**), and F13 to F17 (**D**) *n* = 6.

The percentage releases of mangiferin from the developed nanocrystals [26] were evaluated and are shown in Figure 3. The percentage release of mangiferin varied from 62.45 to 99.02%. The F11 showed the maximum release of mangiferin, and F13 showed the minimum release of mangiferin, as mentioned in Figure 3. The three optimized formulations were developed and showed greater than 90% release of mangiferin, as mentioned in 3. It has been found that the variations in mangiferin concentrations employed in the study are connected to the release profiles. Due to their smaller sizes, the HPMC and pluronic acid may have a greater propensity to aggregate, and the medication was released more slowly from these particles. The values of *R^2^* from zero-order and first-order models ranged from 0.891 to 0.999 and 0.567 to 0.916, respectively, indicating that the developed formulations followed the zero-order release [27]. The value of *n* from the Korsmeyer–Peppas model was below 0.5, indicating the Fickian diffusion of mangiferin release.

### 2.4. Fourier Transform Infrared Spectroscopy (FTIR)

**Figure 4:**
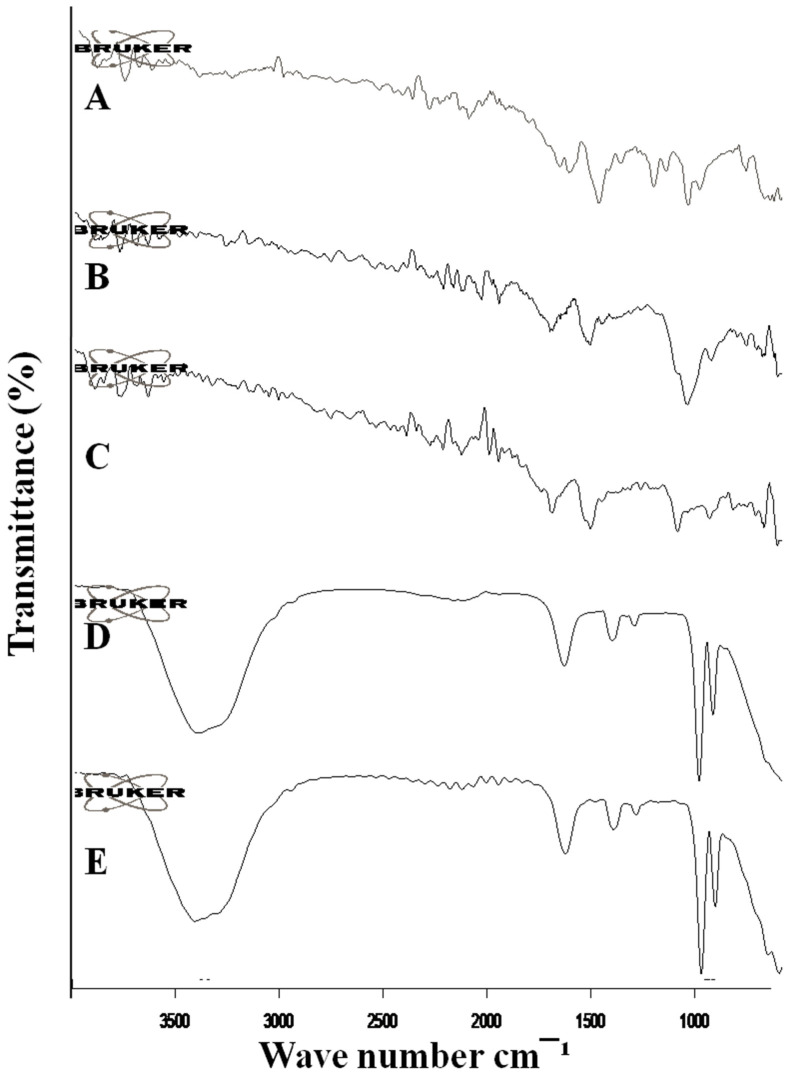
FTIR spectra of mangiferin (**A**), HPMC (**B**), Pluronic F127 (**C**), blank F11 (**D**), and mangiferin-loaded formulation F11 (**E**).

The FTIR spectra of mangiferin, HPMC, Pluronic acid, blank formulation, and mangiferin-loaded formulation are shown in Figure 4. Mangiferin showed characteristic peaks at 3321 cm^−1^, 1510 cm^−1^, and 1086 cm^−1^ due to the presence of –OH bond, C-H, and C-O stretching, respectively [28]. HPMC showed peaks at 1516 cm^−1^ and 1055 cm^−1^ due to the absorption band of C-O ether linkage and stretching of the C-O-C group [29]. The pluronic acid spectra showed peaks at 2127 cm^−1^, 1516 cm^−1^, and 1102 cm^−1^ due to –OH stretching and the CHO group [30]. The unloaded OF1 formulation showed peaks that correspond to the peaks of polymers used for the preparation of nanocrystals. The mangiferin-loaded formulation showed peaks that correspond to the peaks of polymers and the drug, indicating the stability of the formulation, and no chemical interaction was present between polymers and mangiferin.

### 2.5. Differential Scanning Calorimetry (DSC)

**Figure 5:**
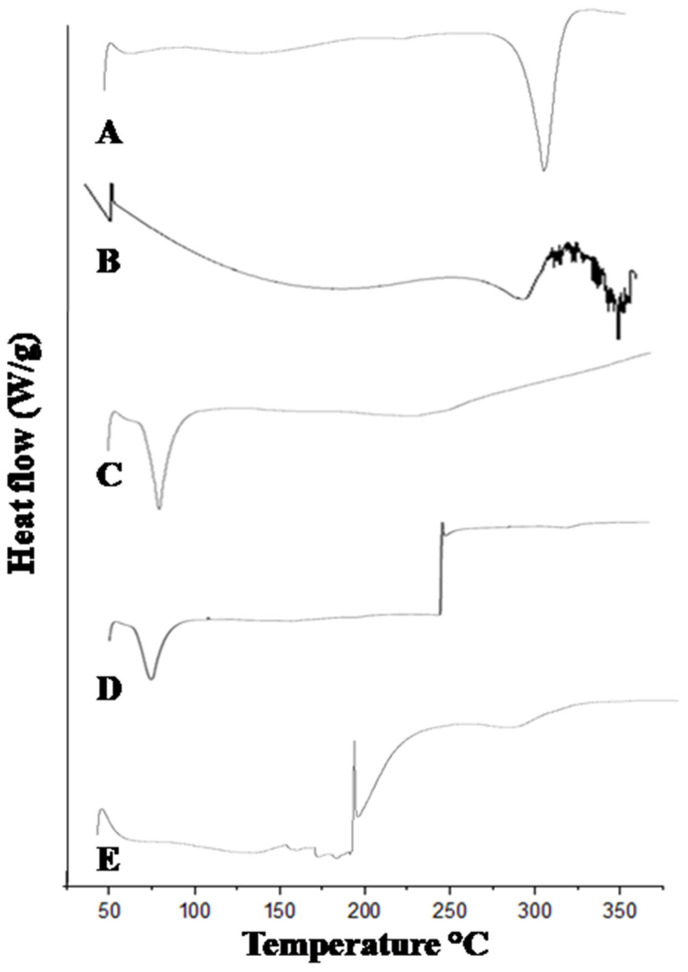
DSC thermogram of mangiferin (**A**), HPMC (**B**), Pluronic F127 (**C**), blank F11 (**D**), and mangiferin-loaded formulation F11 (**E**).

The DSC thermogram of mangiferin showed an exothermic peak at 49 °C and an endothermic peak at 273 °C [31], as shown in Figure 5. Many endothermic peaks were observed in HPMC, while the prominent endothermic appeared at 299 °C [32], as mentioned in Figure 5. Pluronic acid showed endothermic peaks at 62 °C and 199 °C [16]. The mangiferin-unloaded formulation showed endothermic and exothermic peaks at 60 °C and 210 °C, respectively. In mangiferin-loaded formulation, many characteristics of endothermic and exothermic peaks were observed, indicating the successful formation of drug-loaded nanocrystals.

### 2.6. Size of Particles and Zeta Potential

**Figure 6:**
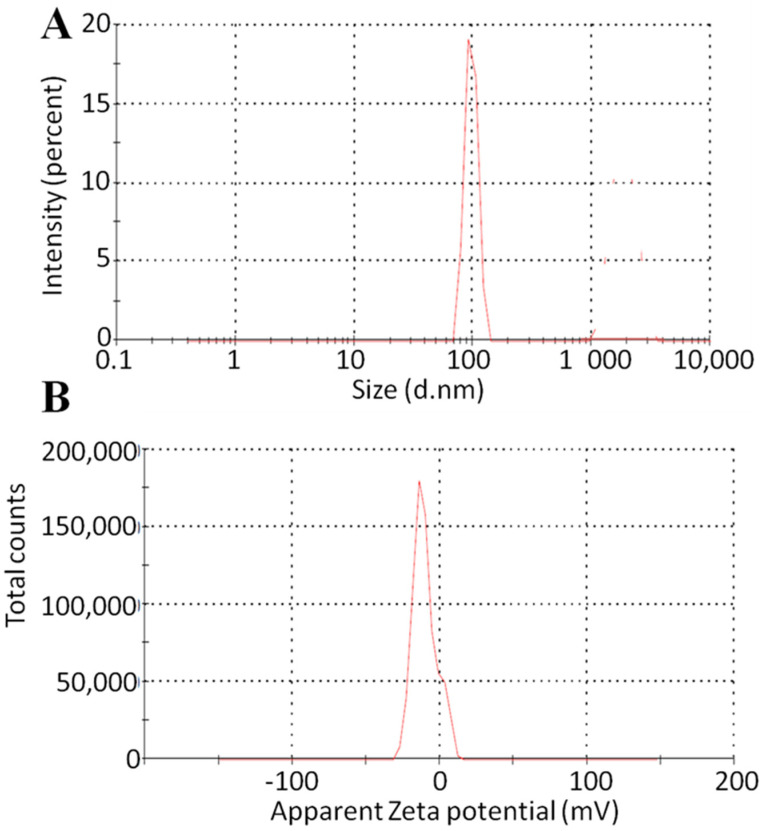
Particle size analysis (**A**) and zeta potential (**B**) of F11 formulation.

The particle size of the various developed and optimized formulations is shown in Figure 6. More than 200 nm particle size was observed in the developed formulation, but the particle size of the optimized formulation was almost 100 nm [33]. The zeta potential values of all developed and optimized formulations were negative. The F11 formulation showed a −12 mV value of zeta potential [34]. As a result, this method demonstrated validity, excellent reproducibility, and suitability for the preparation of nanocrystals. With a size of 100 nm, a PDI of around 0.2, and a zeta potential value of roughly −20 mV, DLS data demonstrated good technological characteristics and predicted strong long-term stability. This might be a result of tween 80 being present on the particle surface and forming a stabilizer layer. Because it regulates the particle size and stability and prevents aggregation during storage, the choice of the surfactant is crucial.

### 2.7. X-ray Diffraction (XRD) Scanning Electron Microscopy (SEM)

**Figure 7:**
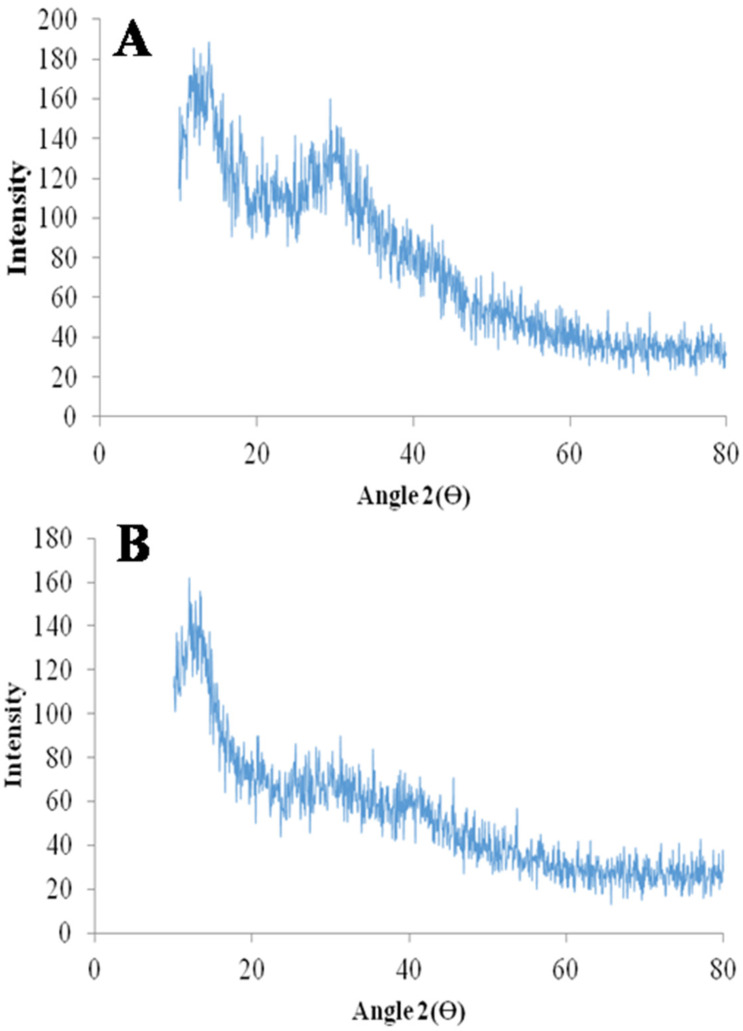
XRD diffractogram of mangiferin (**A**) and mangiferin-loaded (**B**) formulation (F11).

**Figure 8:**
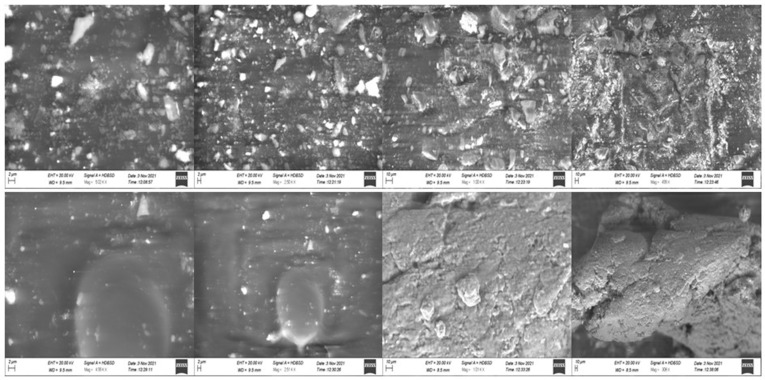
SEM image of OF1 formulation (Blank) at 5.02 KX, 2.50 KX, 1.00 KX, 499 X, and mangiferin loaded at 4.99 KX, 2.51 KX, 1.01 KX, and 308 X.

The XRD diffractogram of mangiferin showed characteristic peaks of diffraction [34,35], and the developed mangiferin-loaded formulation also has diffraction peaks, but the intensity of the peak is less as compared to mangiferin peaks, as mentioned in Figure 7. The intensity of the peak was very low in the mangiferin-loaded formulation, indicating the uniform distribution of the mangiferin in the developed nanocrystals [36]. To ascertain whether the generated particles were crystalline or amorphous, X-ray diffraction examinations on raw and processed samples were conducted. The unprocessed mangiferin peaks demonstrated the compound’s remarkable crystallinity. Although most of the peaks had a lower intensity, the relative amorphization of the mangiferin can be seen in the pattern for nanocrystals. Since the mass transfer between supercritical CO_2_ and the liquid solution of mangiferin occurs quickly in nanocrystals, the molecules do not have enough time to reorganize into a crystalline form during the subsequent precipitation. This circumstance ought to enhance mangiferin solubility in water and simulated fluids. The SEM images of mangiferin-unloaded and -loaded formulation showed nanocrystals, as mentioned in Figure 8. Because the distributions in that technique were larger and the conglomerates of smaller particles were quantified, it is probable that the particle dispersion was adequate.

### 2.8. Stability Studies

The particle size and zeta potential % mangiferin release were evaluated for stability studies, as mentioned in Table 2. A slight change was observed in particle size during the storage of formulation for 6 months. The value of zeta potential was changed from −15 to −09. The % mangiferin release after 6 months was 89.56%.

### 2.9. Pharmacokinetic Study

**Figure 9:**
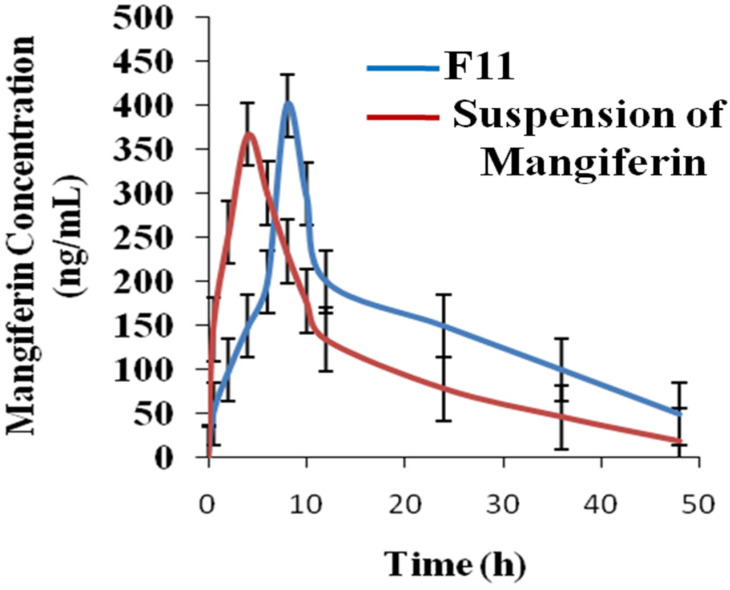
Profile of pharmacokinetics of mangiferin (test and reference formulations) *n* = 6.

The parameters of the pharmacokinetics of mangiferin are mentioned in Figure 9 and Table 3. The peak plasma concentration of mangiferin from nanocrystals (test) and suspension (reference) of mangiferin were 412 ng/mL and 367 ng/mL, respectively [37]. The t_max_ was 8 h and 4 h for the nanocrystals and suspension of mangiferin, respectively. The peak plasma concentration of mangiferin from nanocrystals (test) and suspension (reference) of mangiferin were 412 ng/mL and 367 ng/mL, respectively [38]. The t_max_ was 8 h and 4 h for the nanocrystals and suspension of mangiferin, respectively. The value of t_1/2_ of nanocrystals and suspension of mangiferin was 16.98 h and 11.34 h, respectively, indicating the nanocrystals of mangiferin showed a greater half-life as compared to the suspension of the formulation [39]. The value of AUC_0-t_ of nanocrystals and suspension of mangiferin was 23,567.45 ± 10.876 µg×h/mL and 18,976.12 ± 9.765 µg×h/mL, respectively, indicating the nanocrystals of mangiferin showed greater availability as compared to the suspension of the formulation [40]. The value of AUC_0-∞_ of nanocrystals and suspension of mangiferin was 45,327.60 ± 12.97 µg×h/mL and 32,456.6 ± 12.09 µg×h/mL, respectively, indicating the nanocrystals of mangiferin showed greater availability of mangiferin as compared to the suspension of the formulation. The value of AUMC_0-∞_ of nanocrystals and suspension of mangiferin was 56,784.50 ± 13.567 µg×h/mL and 42,389.45 ± 12.90 µg×h/mL, respectively [41]. The value of MRT of nanocrystals and suspension of mangiferin was 245.23 ± 3.12 h and 176.09 ± 3.09 h, respectively. The value of C_max_ indicated that the availability of mangiferin from nanocrystals was greater as compared to the suspension of mangiferin. AUC values indicated the greater availability of mangiferin from nanocrystals as compared to the suspension of mangiferin.

## 3. Materials and Methods

### 3.1. Materials

Mangiferin was acquired from Xi’an Sgonek Biological Technology Co., Ltd., Beijing, China. Pluronic F127, HPMC K15M, and tween 80 of analytical grade were purchased from Sigma-Aldrich GmbH-Darmstadt, Germany. DMSO, acetonitrile, potassium dihydrogen phosphate, and dichloromethane were obtained from Merck-Darmstadt, Germany. All chemicals were of analytical grade.

### 3.2. Preformulation Studies

Purity of a drug is determined by its melting point. If melting temperature shows variation, then impurities may be present. The melting point of mangiferin was determined. The solubility of mangiferin was determined in different selected solvents. Methanol, ethanol, n-hexane, ether, acetone, and water were used as solvents to check the solubility of mangiferin in different temperature conditions.

### 3.3. Design of Experiments

The fractional factorial design (FFD) [42,43] was used via selection of independent and dependent variables for the preparation of formulations and evaluation of the effect of different excipients on the responses. The selected factors were concentration of mangiferin (A), HPMC (B), pluronic acid (C) tween 80 (D), and ratio of antisolvent to solvent (E). The selected responses were particle size, polydispersity index (PDI), zeta potential, and entrapment efficiency, as mentioned in Table 4.

### 3.4. Preparation of Mangiferin-Loaded Nanocrystals

Seventeen formulations are outlined in Table 5. To prepare these formulations, stock solution of mangiferin of 40 mg/mL was prepared. For this purpose, an accurately weighed amount of mangiferin was dissolved in DMSO in volumetric flask, and the final volume was made up to the mark by the further addition of DMSO. Accurately weighed amounts of polymers, Hydroxypropyl methyl cellulose (Viscosity 2500–5000 Cp), and Pluronic F127 were added into volumetric flask and made the final volume up to mark with the deionized distilled water [44]. The volumetric flask was placed on magnet for two hours for complete mixing of the polymers. Then, tween 80 was added to this anti-solvent mixture. It was cooled in refrigerator. For mixing of both phases, high-speed homogenizer was used at 12,000 rpm for five minutes. Anti-solvent phase (polymer) was placed under the homogenizer, and drug solution was added dropwise during the mixing process. The formed suspension was centrifuged at 16,000 rpm for forty minutes for the separation of nanocrystals. The supernatant was separated carefully. The supernatant was analyzed on UV spectroscopy at 410 nm wavelength, and absorbance was noted. Three readings were performed, and the average absorbance was taken to calculate % entrapment efficiency. The residues collected after the centrifugation were lyophilized after freezing. The powder was used for further analysis. Then, it was freeze dried and further lyophilized into Lyophilizer (Labcono model 195 A 654112906) at −40 °C. The collected powder was labeled and kept for further studies.

### 3.5. HPLC Method Development

Methanol, Acetonitrile, and ortho-phosphoric acid in ratio of 20:40:40 *v*/*v* were used as mobile phase. The flow rate was oscillated from 0.5 to 1.5 mL/min, and the revealing of the mangiferin was made at λ_max_ 258 nm. The mangiferin solution and selected solvents for mobile phase were passed through HPLC system High Performance Liquid Chromatographic system consisting of HPLC pumps (Shimadzu LC-10AT and LC-10AT VP), and a manually operated 20 mL sample loop and UV–Visible detector (SPD 10A VP) was used. The output signal was integrated by Shimadzu class-VP (version 6.12 SP1 software) for 0.5 h to maintain the equilibrium. The mangiferin solution was injected after passing through 0.22-micron filter. The maintenance of system equilibrium and passage of mobile phase through HPLC is necessary.

### 3.6. In Vitro Drug-Release Studies

Using USP-dissolution equipment, an in vitro release analysis of mangiferin from developed nanocrystals was conducted [45]. The dialysis bag method was used to perform in vitro release from unprocessed mangiferin and improved polymeric nanocrystal formulation. As a dissolving medium, pH 7.4 phosphate-buffered solution was utilized. About 50 mg of unprocessed drug and nanocrystals was precisely weighed and placed separately in the dissolving medium. The in vitro release investigations were carried out in 100 mL of release medium at 37 °C with a paddle rotation of 50 rpm. At 0, 0.5, 1, 1.5, 2, 4, and 8 h, an aliquot of the release medium (5 mL) was collected and replaced with an equivalent volume of fresh phosphate buffer, which was pre-warmed at 37 °C to maintain a constant volume of the dissolving medium. The samples were diluted appropriately and passed through a 0.22 µm syringe filter. The drug concentration was subsequently determined using a high-performance liquid chromatographic system (HPLC Perkin Elmer, Flexer) equipped with a stainless steel C18 column (25 cm, 4.6 mm, 5 µm) and a UV/Visible detector at a wavelength of 258 nm. All the experiments were performed in duplicate. The release kinetics of controlled-release polymeric systems was supposed to reflect distinct release processes. As a result, various kinetic models (DD solver) were used to examine the in vitro data to obtain the best-fitting equation.

### 3.7. Fourier Transforms Infrared Spectroscopy (FRIT)

The interaction of mangiferin with pluronic acid and HPMC K15M was studied by FTIR (Bruker Alpha, Bruker Optics, Leipzig, Germany) by observing the peaks of different functional groups. The interaction of different functional groups and peak shifting was observed in order to understand the chemical interaction of drug with the used excipients. The wavelength was recorded in the range of 4000 to 400 cm^−1^.

### 3.8. Differential Scanning Calorimetry (DSC)

The DSC of mangiferin, pluronic acid, HPMC K15M, blank formulation, and mangiferin-loaded formulation was performed at temperature range of 5 to 600 °C with scan rate of 10 °C per minute using Perkin Elmer, 60A, Rodgau, Germany DSC calorimeter.

### 3.9. Zeta Sizer and Zeta Potential

The zeta sizer (Malvern Instruments, Inc., Malvern, Worcestershire, UK) was used to evaluate the size of nanocrystals and surface charge on crystals to investigate the stability of mangiferin nanocrystals.

### 3.10. X-ray Diffraction Analysis (XRD)

The XRD of mangiferin, pluronic acid, HPMC K15M, blank formulation, and mangiferin-loaded formulation was performed at diffraction angle of 0 to 80° (Ɵ) using X-ray diffractometer (D/max-2500pc, Rigaku, Co., Tokyo, Japan).

### 3.11. Scanning Electron Microscopy (SEM)

The surface of prepared nanocrystals was evaluated using SEM analyzer (Hitachi High Tech S4800 FE-SEM) to investigate the surface of mangiferin nanocrystals and size of nanocrystals.

### 3.12. Stability Studies

Optimized formulation (F11) was divided into three portions and put in vials having 15 mL capacity. Aluminum foil was placed on two flasks for the purpose of covering and put at −4 °C in refrigerator. One flask without covering was placed at room temperature. Size of the particle, measurements of surface charge, and release of mangiferin were completed without delay and consequent after 30, 90, and 180 days [46].

### 3.13. Pharmacokinetic Analysis

Rabbits are widely used for in vivo study, as the animal is easy to handle and economical compared to other animals, and the size of a rabbit is greater as compared to rats and mice. The drugs can be administered easily via feeding tube compared to the rats and mice. The withdrawal of sample from the veins of rabbits is easy compared to the rats and mice [47]. 12 Albino rabbits of either sex (age 5–6 months) were taken with a weight range of 2 to 3 kg and divided into 2 equal groups. One group received F11 nanocrystals of mangiferin (test), and second group received suspension of mangiferin (reference). The working procedure and handling of animals were made through the guidelines of ICH after the approval from ethics committee of BZU Multan. Rabbits had complete access to water for 24 h before the dosing but no access to food. The dose containing 0.5 mg/kg body weight was administered using feeding tube [38]. The blood samples having quantity of 0.5 mL were withdrawn from the jugular vein of the rabbit at regular time intervals. Mangiferin was isolated from plasma using extraction method. In summary, 20 µL of plasma was combined with 200 µL of acetonitrile and methanol (50:50 *v*/*v*). After this, the samples were vigorously mixed for 30 s and subjected to centrifugation at 10,000 rpm for 10 min at 4 °C. The resulting supernatant from each sample was transferred carefully to a clean vial. These samples were concentrated by evaporating the solvent under nitrogen at 37 °C and subsequently reconstituted in 100 µL of acetonitrile and methanol (50:50 *v*/*v*). The reconstituted samples were further treated with sonication for 5 min and analyzed using HPLC. The pharmacokinetic parameters are calculated by PK solver Excel-based sheets. PK Solver, a freely available menu-driven add-in program for Microsoft Excel written in Visual Basic for Applications (VBA), was used for solving basic problems in pharmacokinetic (PK) and pharmacodynamic (PD) data analysis. The program provides a range of modules for PK and PD analysis, including non-compartmental analysis (NCA), compartmental analysis (CA), and pharmacodynamic modeling.

## 4. Conclusions

Nanocrystals of mangiferin were developed successfully utilizing pluronic F127, HPMC K15M, and tween 80 using design experts. The factors showed significant effects on the selected responses, and ANOVA results showed the significance of the model. Interaction, contour, and 3D graphs showed the effects of factors on responses. The release of mangiferin from nanocrystals was greater than 85%, indicating good release behavior. FTIR, DSC, XRD, and SEM showed chemical compatibility, thermal stability, reduced crystalline nature of mangiferin in nanocrystals, and surface morphology of developed nanocarriers. The developed nanocrystals of the optimized formulation were 100 nm, and the zeta potential was in the negative range. HPLC method was successfully developed for the quantification of mangiferin in the mobile phase and plasma. The developed nanocrystals showed a stable nature and indicated the improved parameters of pharmacokinetics, such as C_max_, t_max_, and AUC.

Merits and Limitations of the study

- The main advantage of the present research is the enhanced oral bioavailability of mangiferin.
- The reporting of the pharmacokinetics of mangiferin in humans is the major limitation of the research work.

## Figures and Tables

**Table 1:** Observed responses from experimental design.

Code	Particle Size	PDI	Zeta Potential mV	Entrapment Efficiency (%)
F1	475.1	0.565	−0.084	79.46
F2	475	0.587	−14	23.98
F3	456.2	0.498	−6.81	86.23
F4	511.2	0.577	−2.3	72.34
F5	547.7	0.572	−3.87	60.50
F6	418	0.485	−3.88	42.07
F7	429	0.553	−8.28	39.64
F8	480.2	0.563	−17.8	76.83
F9	426.4	0.786	−1.13	51.25
F10	487	0.428	−13	54.44
F11	100.2	0.653	3.08	59.86
F12	620.04	0.709	−1.25	43.74
F13	549.5	0.678	−2.63	50.79
F14	474.7	0.621	−5.21	50.37
F15	549.5	0.678	−2.63	50.09
F16	549.5	0.678	−2.63	50.37
F17	474.7	0.621	−5.21	50.23

**Table 2:** Results of stability studies of mangiferin nanocrystals.

Duration in Months	Particle Size	Zeta Potential	% Mangiferin Release
0	104 ± 1.67	−15	98.23 ± 3.09
1	114 ± 2.34	−11	95.23 ± 3.89
3	115 ± 2.91	−10	92.13 ± 4.67
6	119 ± 3.56	−09	89.56 ± 4.98

**Table 3:** Parameters of mangiferin pharmacokinetics (*n* = 6).

Parameters	Nanocrystals of Mangiferin F11 (Test Formulation)	Suspension of Mangiferin (Reference Formulation)
*t_max_* (h)	8 ± 1.34	4 ± 0.43
*C_max_* (µg/mL)	412 ± 4.10	367 ± 4.23
*t_1/2_* (h)	16.98 ± 2.08	11.34 ± 0.56
AUC_(0−t)_ (µg×h/mL)	23,567.45 ± 10.876	18,976.12 ± 9.765
AUC_(0−∞)_ (µg×h/mL)	45,327.60 ± 12.97	32,456.6 ± 12.09
AUMC (µg×h/mL)	56,784.50 ± 13.567	42,389.45 ± 12.90
MRT (h)	245.23 ± 3.12	176.09 ± 3.09

**Table 4:** Variables and constraints in FFD.

Name	Goal	Lower Limit	Upper Limit	Lower Weight	Upper Weight	Importance
A: Concentration of mangiferin	is in range	20	40	1	1	3
B: Concentration of HPMC	is in range	0	0.5	1	1	3
C: Concentration of Pluronic F127	is in range	0	0.5	1	1	3
D: Concentration of Tween 80	is in range	0.025	0.4	1	1	3
E: Volume ratio of anti-solvent to solvent	is in range	1	9	1	1	3
Particle Size	minimize	200	620	1	1	5
PDI	minimize	0.428	0.786	1	1	3
Zeta Potential	is in range	−17.8	3.87	1	1	3
Entrapment Efficiency	maximize	23.98	86.23	1	1	3

**Table 5:** Composition of formulations with fractional factorial design.

Formulation Code	Conc. of Drug (mg/mL)	Concentration of HPMC (%)	Concentration of Pluronic F127 (%)	Concentration of Tween 80 (%)	Solvent to Anti-Solvent
F1	40.00	0.50	0.50	0.03	1:1
F2	20.00	0.50	0.00	0.03	1:9
F3	40.00	0.00	0.00	0.03	1:1
F4	40.00	0.00	0.50	0.03	1:9
F5	40.00	0.50	0.00	0.40	1:9
F6	20.00	0.00	0.50	0.40	1:1
F7	20.00	0.00	0.00	0.40	1:9
F8	40.00	0.00	0.50	0.40	1:1
F9	20.00	0.50	0.50	0.40	1:9
F10	20.00	0.50	0.00	0.40	1:1
F11	20.00	0.00	0.50	0.03	1:1
F12	40.00	0.50	0.00	0.03	1:9
F13	30.00	0.25	0.25	0.21	1:5
F14	30.00	0.25	0.25	0.21	1:5
F15	30.00	0.25	0.25	0.21	1:5
F16	30.00	0.25	0.25	0.21	1:5
F17	30.00	0.25	0.25	0.21	1:5

## Data Availability

Not applicable.

## Sample Availability

Samples of the compounds are not available from the authors.
